# Evaluation of Pharmacotherapeutic Patterns in Patients With Renal Impairment Undergoing Hemodialysis: A Drug Utilization Study

**DOI:** 10.7759/cureus.99970

**Published:** 2025-12-23

**Authors:** Dilip Kanjariya, Anita Sinha, Hardik Vaniya, Trushti Rathva, Anilkumar Makvana

**Affiliations:** 1 Pharmacology, Government Medical College, Surat, Surat, IND

**Keywords:** acute kidney injury, chronic kidney disease, drug utilization study, hemodialysis, rational prescribing, who prescribing indicators

## Abstract

Background

Chronic kidney disease (CKD) and acute kidney injury (AKI) are significant contributors to renal morbidity and mortality worldwide. In India, the rising prevalence of hypertension and diabetes has led to an increase in CKD cases, with limited access to renal replacement therapies. Hemodialysis remains a common treatment modality, but patients face challenges, including complex medication regimens and adverse drug reactions. Drug Utilization Studies (DUS) are essential to evaluate prescribing patterns and promote rational drug use in this population.

Methodology

A cross-sectional observational study was conducted over 12 months at two setups in Surat, Gujarat, India. Ethical approval was obtained from the HREC. Adult patients (≥18 years) undergoing hemodialysis were enrolled after informed consent. Data were collected using a pre-designed case record form based on WHO core prescribing indicators. Statistical analysis was performed using IBM Corp. Released 2014. IBM SPSS Statistics for Windows, Version 20. Armonk, NY: IBM Corp.

Result

The study was conducted on 150 hemodialysis patients (64% male; mean age 46.5 ± 14.7 years). Most (116; 77.3%) received dialysis 2-3 times per week. Hypertension (97; 64.6%) and diabetes (37; 24.6%) were the leading comorbidities, while 35 (23%) were unaware of their illness. Parenteral medications were prescribed to 104 patients (69.3%), with erythropoietin being the most common (75; 50%). A total of 1130 drugs were prescribed, averaging 7.53 per patient. Out of the total drugs used, 680 (60.17%) were prescribed generically, and 777 (68.76%) were from the National List of Essential Medicines (NLEM). Among drug classes, cardiovascular agents were most frequent (337; 29.8%), followed by gastrointestinal (226; 20%) and hematopoietic agents (112; 12%). Significant differences in drug usage were observed between healthcare setups (p < 0.01).

Conclusion

The study reveals a high prevalence of hypertension and polypharmacy among hemodialysis patients, with significant reliance on cardiovascular and gastrointestinal drugs. Generic and essential drug prescribing rates were suboptimal, and injection use was notably high. Statistically significant differences in drug utilization patterns across healthcare setups underscore the need for rational prescribing and standardization of dialysis pharmacotherapy.

## Introduction

Kidneys are vital organs that maintain the body's chemical balance by removing waste and excess water and regulating essential electrolytes like sodium, potassium, and calcium. Additionally, kidneys produce hormones that stimulate bone marrow to produce red blood cells and help regulate blood pressure. Damage to the kidneys can result from drugs, chemicals, trauma, or genetic factors, leading to renal failure, which can be acute or chronic, depending on the duration and reversibility of the renal damage.

Acute kidney injury (AKI), previously known as acute renal failure (ARF), involves both structural damage and functional impairment. It is characterized by a sudden decline in kidney function, which can occur within hours. Even minor damage, indicated by small fluctuations in urine output and serum creatinine (sCr), can predict severe clinical outcomes [1-3]. Chronic kidney disease (CKD) is characterized by the presence of kidney damage or an estimated glomerular filtration rate (eGFR) of < 60 mL/min/1.73 m², persisting for ≥3 months, irrespective of the cause [4]. CKD is a major public health concern globally, associated with high morbidity and mortality [2,5]. It often progresses to end-stage renal disease (ESRD), which necessitates renal replacement therapy, such as transplantation or dialysis [4]. The primary causes of ESRD include complications of uncontrolled hypertension and diabetes, while other causes can be renal (glomerulonephritis, focal-segmental glomerulosclerosis), post-renal (reflux nephropathy, obstruction), or pre-renal (chronic or acute ischemia) [6].

In India, the increasing prevalence of hypertension and diabetes has led to a significant rise in CKD cases. The limited availability of healthcare facilities, inadequate management of risk factors, and late referrals to specialists contribute significantly to the progression of CKD to ESRD. In 2005, it was estimated that the prevalence of stage 3 and higher CKD was 7,852 cases per million people (0.785%) [7]. In 2018, it was estimated that around 175,000 patients in India were undergoing chronic dialysis, corresponding to a prevalence of 129 per million population [8]. The financial burden associated with renal disease management prevents more than 90% of patients from receiving adequate treatment [2]. Kidney transplantation aims to provide patients with ESRD a longer and better quality of life. The two primary methods for transplantation and procurement are open surgery and laparoscopic surgery [6]. However, due to the high cost of transplantation, most patients opt for dialysis as a more affordable alternative. There are two main types of dialysis: hemodialysis and peritoneal dialysis. Hemodialysis involves circulating a patient's blood extracorporeally through an artificial kidney to exchange solutes. Peritoneal dialysis, on the other hand, removes waste and excess fluid by introducing a fluid into the patient's abdominal cavity via a peritoneal dialysis catheter [9].

Patients on chronic hemodialysis face numerous challenges, including medication adherence, which can lead to adverse drug reactions and a decline in quality of life [10,11]. Rationalizing drug prescriptions for CKD patients is essential to minimize side effects and provide optimal care. This is particularly challenging due to the complex therapeutic regimens required and the presence of comorbidities such as hypertension, coronary artery disease, infections, and diabetes mellitus [11]. Inappropriate drug use can exacerbate negative effects, leading to longer hospital stays, increased healthcare consumption, and higher costs [11].

The World Health Organization (WHO) defines a drug utilization study (DUS) as the marketing, distribution, prescription, and use of drugs in society, with a focus on the resulting medical, social, and economic consequences [12]. Accurate data interpretation at the patient level is crucial for rational drug use, allowing for the formulation of regulations and policies based on DUS findings [12].

To address these challenges, a drug utilization study was planned in South Gujarat.

## Materials and methods

Ethical clearance

This cross-sectional observational study was approved by the HREC, Government Medical College Surat, under approval No. GMCS/STU/ETHICS-3/Approval/8962/23 on August 1st, 2023. The study began upon receipt of the approval letter and was conducted over one year, from August 1, 2023, to July 31, 2024.

Sample size

A sample size of 150 was calculated using the following formula: 

$n=\frac {[DEFF*Np(1-p)]} {[(d2/Z21-&alpha;/2*(N-1) +p*(1-p)]}$

where a design effect (DEFF) of 1 was used because random sampling was employed. The total population size (N) was 243 based on previous data from the study site. An anticipated frequency (p) of 50% and a Z value of 1.96, corresponding to a 95% confidence level, were incorporated into the calculation.

Selection criteria

Inclusion Criteria

Participants eligible for the study were adults aged 18 years or older of either gender who were undergoing hemodialysis, irrespective of the frequency or duration of their treatment.

Exclusion Criteria

Patients with a prior or current history of psychiatric illness, those with incomplete case records, or patients who refused to give consent were excluded.

Hemodialysis patients at a tertiary care hospital (Setup 1) and a nonprofit facility located within a tertiary care hospital campus (Setup 2) were selected as study participants. Participants were invited to participate in this study and given participant information sheets explaining their duties. These were given to all patients as per the inclusion and exclusion criteria who had a clinician-diagnosed need for hemodialysis. After their consent was obtained, those who agreed to take part in the study were given an informed consent form. Each subject gave written informed consent before being enrolled in the study. Data was collected from each patient only once, and no patient was repeated.

The drug utilization pattern was assessed using a pre-designed case record form based on the WHO core prescribing indicators [12] form for all patients irrespective of the time duration of treatment. This record documented pharmacotherapeutic parameters such as the average number of drugs prescribed per patient, the proportion of prescriptions containing antibiotics or injections, the percentage of drugs prescribed using generic names, and the percentage of drugs selected from the National List of Essential Medicines (NLEM) 2022 [13].

Statistical analysis

Data were analyzed using percentages and frequencies, the chi-square test was applied for checking association, and proportions were applied for categorical data. The study used IBM Corp. Released 2014. IBM SPSS Statistics for Windows, Version 20. Armonk, NY: IBM Corp. for the analysis.

## Results

Demographic and clinical profile

A total of 150 patients undergoing hemodialysis were enrolled in this study, including 84 patients from setup 1 and 66 patients from setup 2. Out of 150 patients, 96 (64%) were male, and 54 (36%) were female. The majority of patients were aged between 31 and 60 years, with a mean age of 46.5 ± 14.7 years. A total of 116 patients (78%) were undergoing hemodialysis 2 to 3 times per week. Only 4% (six patients) were undergoing dialysis once per week. The rest were AKI patients.

**Figure 1:**
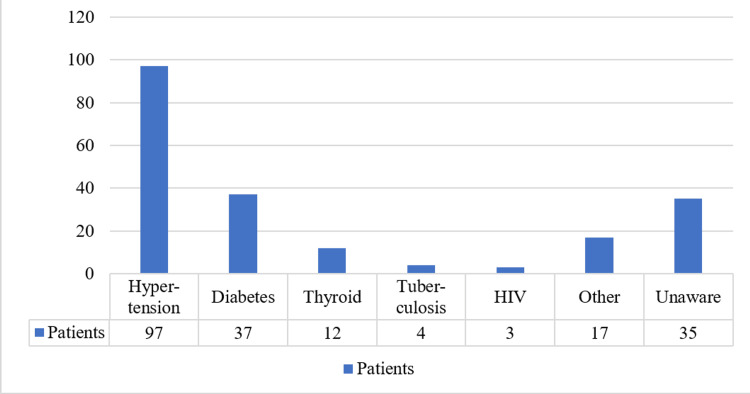
Patients with co-morbidities

Considering comorbidity, 97 patients were suffering from hypertension, and 37 patients were suffering from diabetes mellitus as a comorbidity. Thyroid/parathyroid diseases, liver disease, gout, cancers, asthma, heart disease, tuberculosis, HIV, and immune thrombocytopenic purpura were the other comorbidities noted in participants. Interestingly, 23% of patients were unaware of their comorbid condition, but most of them were on antihypertensive therapy (Figure 1).

WHO prescribing indicators

Table 1 shows a total of 1130 drugs being prescribed from the 150 analyzed prescriptions. The studied centers had an average of 7.53 (SD=3.86) drugs prescribed per consultation, and an average of 60.17% (N=680) were prescribed by generic name. Additionally, 25.33% (N=286) of all encounters were prescribed antibiotics during the reviewed consultations. On average, 68.76% (N=777) of the antibiotics appeared on the NLEM. 104 patients (69.33%) were prescribed one or more parenteral drugs.

**Table 1:** WHO prescribing indicators *WHO prescribing indicators and optimal values [14,15]

WHO core prescribing indicators*	Calculated Values	Optimal value*
Total number of drugs	1130	
Average number of drugs per prescription	7.53	1.6-1.8
Percentage of drugs prescribed by generic name	60.17%	100%
Percentage of drugs prescribed from essential medicine list	68.76%	100%
Percentage of patients with an injection prescribed	69.33%	13.4-24.1%
Percentage of patients prescribed with antibiotics	25.33%	20.0-26.8%

Prescription patterns in acute vs. chronic kidney disease patients

Table 2 provides a detailed comparison of prescription patterns in acute and chronic patients on dialysis, highlighting the total number of drugs prescribed, the number of fixed-dose combinations, generic drugs, essential drugs, and antibiotics used. 

**Table 2:** Prescription patterns in acute vs. chronic kidney disease patients FDC: Fixed-dose combination

	Total	Acute	Chronic
Prescription analyzed	150	28 (18.67%)	122 (81.33%)
Total number of drugs prescribed	1130	253 (22.4%)	877 (77.6%)
Number of FDC Prescribed	104	21 (20.2%)	83 (79.8%)
Number of generic drugs Prescribed	680	156 (22.9%)	524 (77.1%)
Number of essential drugs Prescribed	777	187 (24.1%)	590 (75.9%)
Antibiotics usage	57	19 (33.3%)	38 (66.6%)

Drug utilization according to the class of drugs 

**Figure 2:**
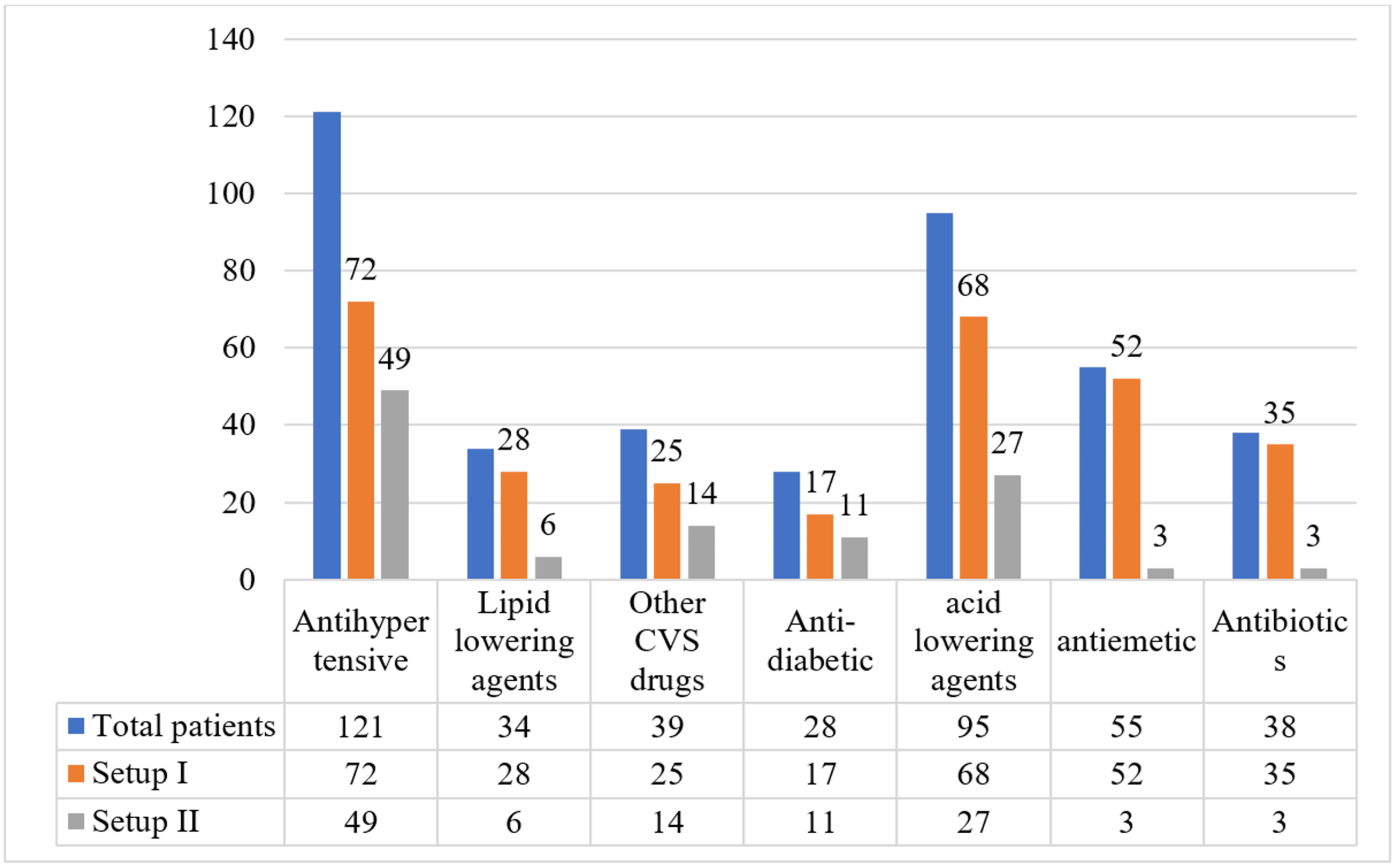
Drug utilization according to class of drugs Setup I: Tertiary care hospital, Setup II: Nonprofit dialysis center, CVS: Drug usages for cardiovascular conditions

Figure 2 shows the number of patients receiving drugs for different health conditions from 150 encounters. Out of 150 patients, 121 (80.7%) patients were on antihypertensive drugs, and 95 (63.3%) patients were on drugs that lower acid secretion.

Drug usage pattern in patients on maintenance hemodialysis

As the majority of patients were receiving antihypertensive therapy, drugs used for cardiovascular conditions represented the most frequently prescribed group, accounting for 29.8% of all medications. This was followed by drugs used for gastrointestinal conditions, which comprised 20%, and medications prescribed to enhance hematopoiesis, which accounted for 12%. The 10 most commonly utilized drugs or drug classes included calcium channel blockers (8.4%), proton pump inhibitors (8%), calcium carbonate and calcium acetate preparations (7%), erythropoietin (6.6%), α2-agonists (5.9%), multivitamin and multimineral supplements (5.6%), antiemetic medications (5.1%), antibiotics (5%), sodium bicarbonate (4.3%), and diuretics (4.2%). Among these 10 prescribed drug classes, three belonged to the anti-hypertensive category (Table 3).

**Table 3:** Drug usage pattern in patients on maintenance hemodialysis ACE: Angiotensin-converting enzyme, ARB: Angiotensin receptor blocker, FDC: Fixed-dose combination

Types of Drugs	Number of Drugs (%)
Drugs used for Cardiovascular conditions	337(29.8%)
Calcium channel blockers	95(8.4%)
Alfa 2 agonist	67(5.9%)
Diuretics	47(4.2%)
Anti platelet	37(3.3%)
Lipid lowering	34(3%)
Beta blocker	28(2.5%)
Alfa + beta blocker	7(0.6%)
Alfa‑blocker	6(0.5%)
ACE inhibitor & ARB	4(0.4%)
Other	12(1.1%)
Drugs used for conditions of gastrointestinal system	226(20%)
Proton pump inhibitors	90(8%)
Antiemetic	58(5.1%)
Sodium bicarbonate	49(4.3%)
Laxatives	17(1.5%)
H2 blockers	8(0.7%)
Anti spasmodic	3(0.3%)
Other antacid	1(0.1%)
Antidiabetics	38(3.4%)
Insulin	20(1.8%)
Metformin	3(0.3%)
Other combined oral hypoglycaemic drugs (FDC)	15(1.3%)
Hematopoietic drugs	136(12%)
Erythropoietin	75(6.6%)
Iron	34(3%)
Folate	22(1.9%)
Other	5(0.4%)
Phosphate binders	109(9.6%)
Calcium carbonate & acetate	79(7%)
Sevelamer	30(2.7%)
Vitamins and minerals	107(9.5%)
Vitamin D3	42(3.7%)
Calcitriol	2(0.2%)
Multivitamins and multimineral	63(5.6%)
Antibiotics	57(5%)
Cefixime	11(1%)
Ceftriaxone	9(0.8%)
Metronidazole	4(0.4%)
Levofloxacin	3(0.3%)
Other antibiotics	30(2.7%)
Other	120(10.6%)
Total	1130

Evaluation of drug utilization differences between two healthcare setups

Table 4 presents data on drug usage across different therapeutic categories in two setups: Setup I and Setup II. The categories include drug usages for cardiovascular conditions (CVS), drugs used for conditions of the gastrointestinal system (GIT), hematopoietic supplements, phosphate binders, and vitamin and mineral supplements. Out of the total prescribed drugs, the maximum number of drugs (N=337) were prescribed for cardiovascular conditions, followed by 226 drugs prescribed for GIT conditions.

**Table 4:** Comparison of drug use between different healthcare setups *A p-value of <0.05 is considered statistically significant. Setup I: Tertiary care hospital, Setup II: Nonprofit dialysis center, CVS: drug usages for cardiovascular conditions, GIT: drugs used for conditions of the gastrointestinal system

	CVS	GIT	Hematopoetic supplement	Phosphate binders	Vitamins & minerals	Total drug usage	chi-square statistic	p-value
Setup I	221	179	75	77	91	643	38.2653	<0.00001*
Setup II	116	47	61	32	16	272		
Total	337	226	136	109	107	915		

The chi-square statistic of 38.2653 and a p-value of less than 0.01 indicate that there is a statistically significant difference in drug usage between Setup I and Setup II across all categories.

Utilization of drugs by route of administration

Various systemic routes were used in drug administration: 1) oral, 2) inhalational, and 3) parenteral. A maximum of 81.68% of drugs were given by oral route, followed by 17.96% by parenteral route and 0.35% of drugs by inhalational route out of a total of 1130 prescribed drugs. Parenteral drugs were further divided into subclasses.

Table 5 shows the distribution of 203 parenterally utilized drugs among 104 patients of hemodialysis, with percentages as well as the number of patients. A total of 104 patients (69.33%) were prescribed at least one parenteral drug.

**Table 5:** Parenteral drug distribution

Parenteral drugs	Number of Drugs (%)	Patients
Intravenous	131 (11.59%)	70
Intramuscular	13 (1.15%)	10
Subcutaneous	59 (5.22%)	53

Table 6 shows the different types of parenteral drugs administered to patients along with their corresponding numbers and percentages. Erythropoietin is the highest administered parenteral drug, accounting for 50% of the patient’s usage, followed by antibiotics and vitamin B12 supplements. Parenteral pantoprazole was mostly given as an adjunct to intravenously administered antibiotics, where oral drug administration was not possible. The data highlights the significant use of erythropoietin and the varied necessity for other parenteral preparations like iron and vaccines in patients.

**Table 6:** Parenteral drug utilization Inj.: Injection

Injections	Number of patients	Percentage
Inj. Erythropoietin	75	50
Injectable Antimicrobials	29	19.3
B12 and other supplements	22	14.7
Antidiabetics (Insulin)	20	13.3
Inj. Iron	18	12
Inj. Pantoprazole	14	9.3
Vaccine	8	5.3

Use of supplements

**Figure 3:**
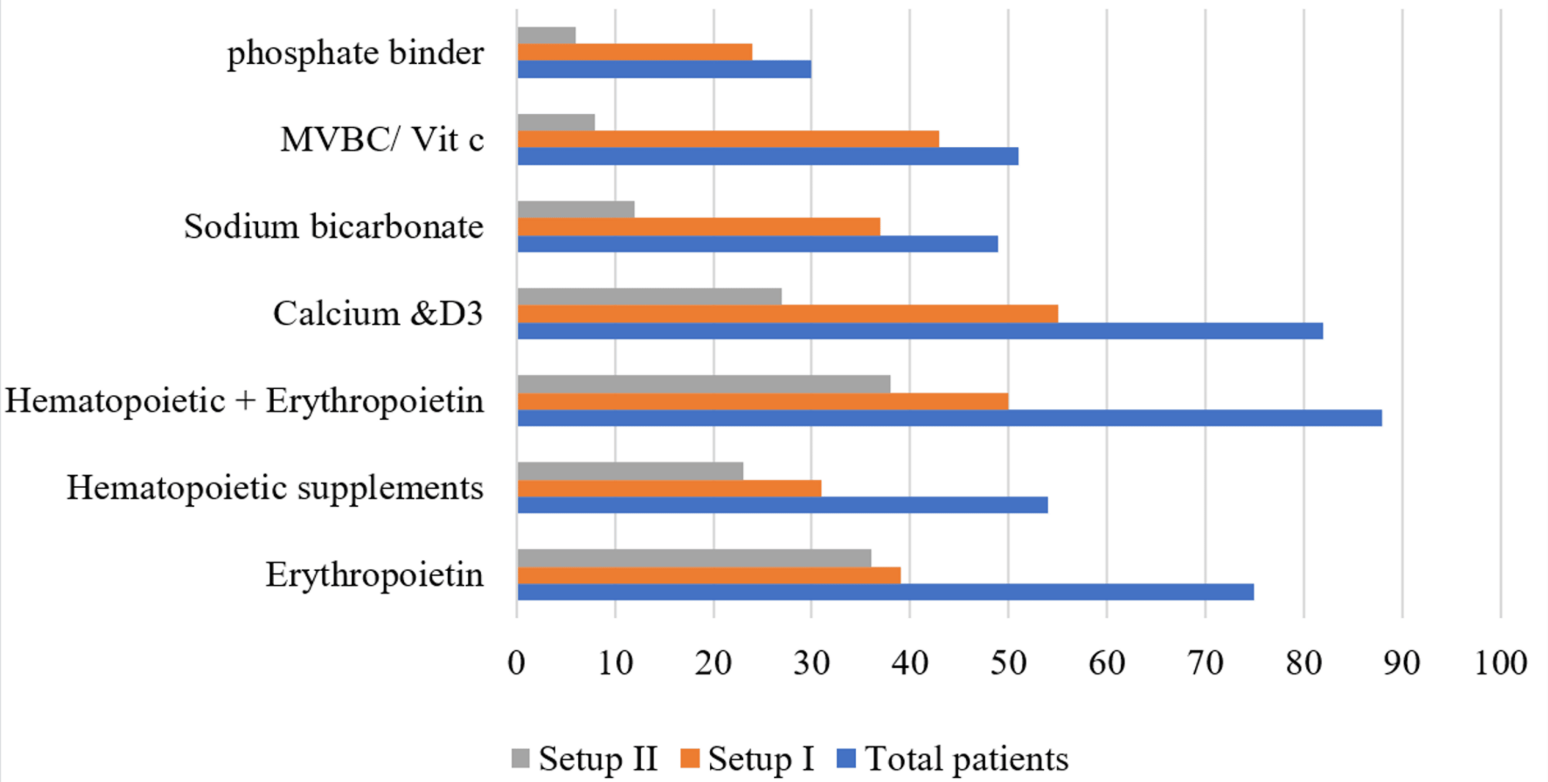
Supplement use in individual setup Setup I: Tertiary care hospital, Setup II: Nonprofit dialysis center, MVBC: Multivitamin B Complex, Vit C: Vitamin C, D3: Vitamin D3

Figure 3 shows data on the usage of supplements for treatments in Setup I and Setup II. The treatments included erythropoietin, hematopoietic supplements, hematopoietic supplements + erythropoietin, calcium and vitamin D3, sodium bicarbonate, MVBC/vitamin C, and phosphate binders.

## Discussion

The present cross-sectional study highlights the complexity of drug utilization in patients undergoing hemodialysis. The frequent use of multiple drugs among these patients reflects the burden of comorbid conditions and complications associated with chronic kidney disease (CKD). Previous researchers have suggested that the overall number of drugs prescribed may serve as an indirect measure of frailty, and our findings support this interpretation [10,16].

Male predominance, along with hypertension, diabetes mellitus, and cardiac diseases as leading comorbidities, is consistent with earlier Indian studies [17]. Notably, 23% (N=35) of patients were unaware of their comorbid status despite being on antihypertensive treatment, underscoring the need for improved patient education. Of these, 22 patients were aged between 30 and 50 years.

In women of reproductive age, acute kidney injury following HELLP syndrome, seizures, or infections progressed to CKD, highlighting obstetric complications as an important contributor to renal morbidity [18].

A total of 81 patients (54%) were on dialysis for less than 12 months, with declining numbers as duration increased. This trend reflects mortality associated with CKD, influenced by cardiovascular events, infections, noncompliance, high treatment costs, poor quality of life, lack of family support, electrolyte imbalance, renal transplantation, and limited availability of dialysis facilities [6,19]. Use of cardiovascular drugs is highest in recent studies by Sridharan et al. and Kirchmayer et al. [20,21].

Importantly, 57% of patients adhered to the recommended thrice-weekly dialysis schedule of 4-hour sessions [22], which is substantially higher than the 20% reported in previous Indian studies [23]. This improvement may be attributed to better accessibility and government support schemes.

Drug utilization analysis revealed a mean of 7.53 drugs per patient, comparable to Tozawa et al. [10]. People with diabetes were given more medicines, which supports the findings of Manley et al. [24]. Mortality has been shown to be higher in patients prescribed more than seven drugs, emphasizing the risks of polypharmacy [10]. Polypharmacy can be reduced by regular medication review, adherence to evidence-based treatment guidelines, and de-prescribing of unnecessary or duplicate therapies. Prescribing indicators revealed suboptimal use of generic names (60.17%) and essential medicines (68.76%) as compared to optimal values [13]. The high rate of parenteral prescriptions (69.33%) was largely due to erythropoietin, which is essential in anemia management.

Significant differences in drug utilization were observed between the two setups (p < 0.05). Setup I showed higher use of cardiovascular and gastrointestinal drugs, likely due to their role in managing critically ill patients. Setup II had lower supplement use, reflecting different healthcare priorities to use their resources to provide supplements. Gastrointestinal drugs, particularly proton pump inhibitors and H2 receptor antagonists, were widely prescribed, consistent with the high prevalence of gastrointestinal complications in hemodialysis patients.

The majority of drugs were administered orally, yet parenteral therapy was notable, being given to 69.33% of patients. Erythropoietin was the most frequently used parenteral drug, prescribed to half of the study population. Antibiotics and vitamin B12 supplements were also commonly used, reflecting their importance in patient management. Pantoprazole was primarily administered parenterally alongside intravenous antibiotics, highlighting the need for alternative routes when oral intake was not feasible. Additional parenteral agents such as iron and vaccines were used, indicating diverse therapeutic requirements among hemodialysis patients.

Patients on hemodialysis often develop anemia due to reduced erythropoietin production. Parenteral erythropoietin, which stimulates red blood cell formation, was used in 50% of cases in this study, with slightly higher usage in Setup II (54.6%) compared to Setup I (46.4%). Other hemopoietic agents, such as iron and folic acid, were more frequently used in Setup I. Erythropoietin administration was guided by hemoglobin levels; previous studies also reported variable erythropoietin usage patterns [25,26]. Supplement use varied between setups, with calcium and vitamin D3, sodium bicarbonate, and multivitamins showing significant differences. Sevelamer was prescribed in 20% of patients, lower than in Farooqui et al. [25,26] but similar to Chakraborty et al. [26].

Overall, this study underscores the challenges of polypharmacy, anemia management, and adherence to dialysis schedules in CKD patients. Indian Government scheme Pradhan Mantri Jan Arogya Yojana (PM-JAY) has improved access to dialysis by providing financial support to patients for diagnosis, treatment, and travelling. However, rational prescribing and patient education remain critical areas for intervention. 

Limitations

This study is limited by its small sample size, which reduces external validity and restricts extrapolation to broader hemodialysis populations. The single-center design, despite the inclusion of government and charitable trust settings, constrains generalizability to private institutions with differing demographics and prescribing practices. Its cross-sectional nature precludes causal inference and fails to capture longitudinal pharmacotherapeutic trends, while the one-year duration is insufficient to assess long-term variability in drug utilization among chronic hemodialysis patients.

## Conclusions

This study emphasizes the multifaceted challenges encountered by patients undergoing hemodialysis, including the burden of multiple comorbidities, extensive drug use, and elevated mortality. Hypertension, diabetes mellitus, and cardiovascular diseases emerged as the predominant causes of CKD, highlighting the importance of early detection and effective management strategies. Polypharmacy, particularly in diabetic patients, was frequently observed (≥8 drugs per patient), raising concerns about drug interactions and treatment adherence. The findings provide valuable insights into drug utilization patterns in this population. Strengthening access to essential medicines, ensuring adherence to recommended dialysis schedules, and improving patient support systems are key measures that can enhance outcomes for individuals on hemodialysis.
